# Supplementary material for: Complexome profiling of the Chlamydomonas psb28 mutant reveals TEF5 as an early PSII assembly factor
Source: Plant Cell. 2025 Mar 17;37(6):koaf055. doi: 10.1093/plcell/koaf055 (PMC12164590; doi:10.1093/plcell/koaf055)
Supplement: koaf055_Supplementary_Data [file koaf055_supplementary_data.zip › psb28_interactive.zip!/plots/intensity/qualityPlots/peptides/ZNJ1_Cre11.g475850.t1.2.html]

### Cre11.g475850.t1.2

Trivial name: ZNJ1  
SeqCoverage [median]: 13.80  
Mass: 21.11 kDa  
  
 

| Sequence | MissedCleavage | Mass [Da] | wtIntensity | muIntensity | Score |
| --- | --- | --- | --- | --- | --- |
| MQTGELKPGEVQDLLTK | 1 | 1885.98 | 0.00 | 5361.85 | 48.88 |
| RPEASLLVQK | 1 | 1139.67 | 0.00 | 2387.58 | 84.21 |
|  |  |  | 0.0 | 7749.4 |  |
